# Percutaneous Transaxillary Access for Large‐Bore Cardiovascular Interventions: Evidence, Technique, and Complication Management

**DOI:** 10.1002/ccd.70759

**Published:** 2026-07-21

**Authors:** Gioel Gabrio Secco, Marco Lombardi, Edoardo Elia, Juan Guido Chiabrando, Andrea Demarchi, Rocco Vergallo, Andrea Audo, Giulia Maj, Giuseppe Patti, Nicolas M. Van Mieghem

**Affiliations:** ^1^ Interventional Cardiology Unit, AOU SS Antonio e Biagio e Cesare Arrigo Alessandria Italy; ^2^ Department of Translational Medicine University of Eastern Piedmont Novara Italy; ^3^ Interventional Cardiology Unit, UOSD Emodinamica, Ospedale Barone Romeo Patti Italy; ^4^ Department of Internal Medicine and Medical Specialties (DIMI) Università di Genova Genova Italy; ^5^ Cardiothoracic and Vascular Department (DICATOV) IRCCS Ospedale Policlinico San Martino Genova Italy; ^6^ Division of Cardiac Surgery, AOU SS Antonio e Biagio e Cesare Arrigo Alessandria Italy; ^7^ Division of Cardiothoracic Intensive Care, AOU SS Antonio e Biagio e Cesare Arrigo Alessandria Italy; ^8^ Division of Cardiology, Maggiore della Carità Hospital Novara Italy; ^9^ Department of Cardiology Thoraxcenter, Erasmus University Medical Center Rotterdam the Netherlands

**Keywords:** large‐bore arterial access, mechanical circulatory support, percutaneous transaxillary access, TAVR, vascular closure

## Abstract

Percutaneous transaxillary access is an established alternative to transfemoral large‐bore arterial access for transcatheter structural heart interventions and mechanical circulatory support (MCS). Although useful for overcoming hostile iliofemoral anatomy and providing prolonged support and patient mobilization in case of MCS, the axillary/subclavian territory poses distinct technical and anatomical challenges that demand meticulous pre‐procedural imaging, patient selection, accurate puncture under ultrasound and angiographic guidance, preservation of bailout access, and familiarity with suture‐based and balloon‐assisted closure strategies. This review provides a contemporary practical framework for percutaneous transaxillary access, summarizing the available clinical evidence, technical aspects of puncture and closure, complication management, and procedural tips to improve safety and reproducibility.

**Abbreviations**
CT: computed tomography
HR‐PCI: high‐risk percutaneous coronary intervention
MCS: mechanical circulatory support
PAD: peripheral arterial disease
PCI: percutaneous coronary intervention
TAVR: transcatheter aortic valve replacement
Tax: transaxillary
VARC: Valve Academic Research Consortium
VCD: vascular closure device

## 1 Introduction

Large‐bore arterial access has become a cornerstone of contemporary interventional cardiology, driven by the rapid expansion of transcatheter structural heart interventions and mechanical circulatory support (MCS) [1, 2]. Although the common femoral artery remains the preferred access site for procedures such as transcatheter aortic valve replacement (TAVR) and MCS [3], a substantial proportion of patients present with calcific or atherosclerotic iliofemoral arterial disease, vessel tortuosity, or small‐caliber vessels that preclude safe transfemoral access [4]. In TAVR populations, alternative access routes are required in approximately 15–20% of patients [5]. Among the available alternatives, the axillary artery has emerged as one of the most attractive access sites [6].

The transaxillary access (TAx) has evolved from a mandatory surgical cutdown to a completely percutaneous technique, using direct puncture and suture‐mediated closure devices [7]. Since then, improvements in imaging guidance, closure technologies, and operator experience have facilitated broader adoption of this strategy [7, 8, 9, 10, 11, 12, 13].

The aim of this review is to provide a contemporary and pragmatic overview of completely percutaneous TAx (pTAx) for structural heart interventions and MCS implantation, from patient selection and arterial puncture to closure, complication management, and practical tips to improve procedural safety (Central Illustration 1).

**Central Illustration 1:**
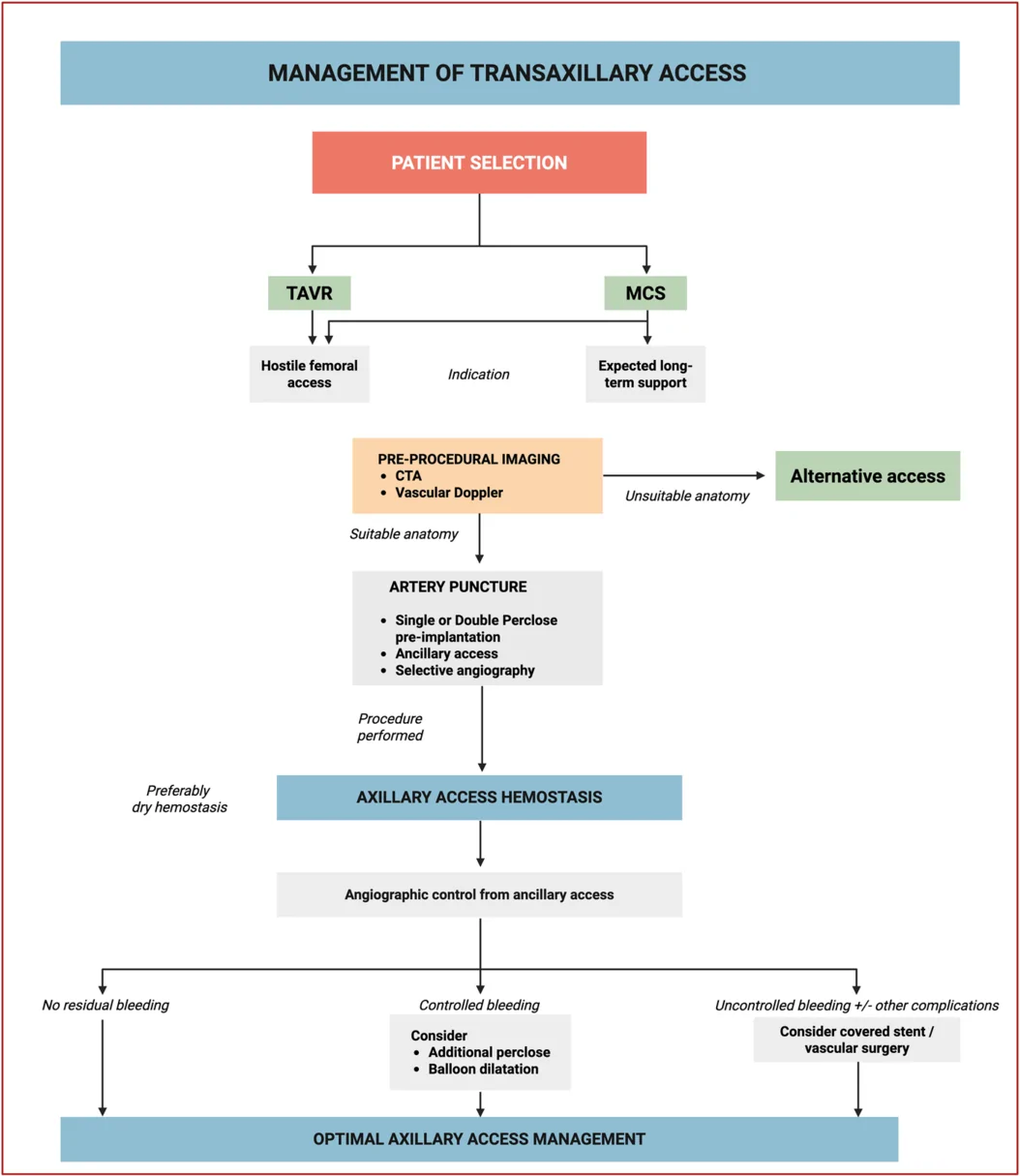
Management of transaxillary access step‐by‐step. [Color figure can be viewed at wileyonlinelibrary.com]

### 1.1 Rationale for Transaxillary Percutaneous Access (pTAx)

Although contemporary management of patients with a hostile ilio‐femoral axis increasingly incorporates endovascular techniques—including balloon angioplasty, intravascular lithotripsy, and stenting—to facilitate device advancement, cases in which this approach carries a prohibitive risk of complications may warrant consideration of an alternative, non‐femoral access site by a multidisciplinary team [13, 14]. Several alternative routes for large‐bore cardiovascular interventions are available, including transapical, transaortic, transcarotid, transcaval, and transaxillary approaches [15]. As transapical and transaortic approaches were associated with an increased risk of mortality compared to the transfemoral approach or conventional surgery [16, 17], the axillary artery has emerged as an attractive option because it combines several practical and anatomical advantages [18, 19, 20, 21, 22].

First, TAx preserves a fully percutaneous strategy, avoiding the surgical exposure required for transthoracic or apical approaches; second, the axillary artery typically provides adequate vessel caliber and relative resistance to advanced atherosclerosis, making it suitable for the introduction of contemporary large‐bore sheaths. Finally, the short and relatively straight trajectory to the ascending aorta facilitates device delivery and coaxial alignment during structural interventions. Importantly, the proximal segment of the axillary artery can be punctured over the rib cage, allowing potential external compression or endovascular hemostasis in case of bleeding complications and precluding hemothorax. These anatomical characteristics, combined with increasing operator experience and advances in percutaneous closure techniques, have supported the development of completely pTAx strategies.

### 1.2 Safety and Efficacy of PTAx

Patients undergoing pTAx represent a particularly high‐risk subgroup, typically characterized by severe peripheral arterial disease (PAD), hostile iliofemoral anatomy, and an increased burden of baseline comorbidity. Accordingly, the elevated incidence of procedural and periprocedural complications should be interpreted in the context of this unfavorable preprocedural risk phenotype rather than related to the access per se [16, 18, 21, 23].

### 1.3 Evidence From Registries and Observational Studies

Across TAVR observational studies and registries, pTAx access has shown high technical success with acceptable rates of vascular and bleeding complications when performed in experienced centers [24].

A multicenter registry from the United Kingdom including 210 patients undergoing pTAx TAVR reported Valve Academic Research Consortium 3 (VARC‐3) technical success in 92.4% of cases. Major vascular access‐site complications occurred in 7.1%, major or life‐threatening bleeding in 5.3%, covered stent use in 25.2%, vascular surgical intervention in 3.3%, in‐hospital stroke in 7.1%, and in‐hospital mortality in 3.3%. These findings highlight the feasibility of the percutaneous approach while emphasizing the residual risk of neurological events and the need for improved access management [25].

In a single‐center observational study of 2,164 TAVR procedures, 60 were performed through TAx because transfemoral access was not feasible. Despite a higher‐risk baseline profile in the TAx cohort, procedural success and 30‐day outcomes including mortality, stroke, major vascular complications, and major bleeding were not significantly different from the transfemoral group [26].

The multicenter NORTHOSTAVI registry enrolled 531 patients treated through alternative access routes for TAVR. pTAx access accounted for 29% of cases and achieved technical success rates of approximately 95%. Thirty‐day overall and event‐free survival were comparable to other endovascular approaches, although the risk of stroke was higher [21]. Notably, when compared to trans‐carotid access, patients in the pTAx group underwent more frequent pre‐ and post‐dilatation, likely due to the greater use of the transcatheter valve, which may have contributed to the increased incidence of cerebrovascular events. Conversely, large‐bore catheter manipulations in the subclavian and axillary arteries may affect ipsilateral brain perfusion and result in debris embolization.

Additional insights are provided by the Hostile registry, a large international study including 1,707 patients with severe PAD and hostile femoral anatomy undergoing TAVR [18]. Among these patients, 547 were treated through non‐femoral transarterial access routes (non‐thoracic access), of which 92% were performed via the TAx approach. Both transfemoral access after peripheral intervention and non‐femora transarterial access were associated with significantly lower 30‐day major adverse event rates compared with transthoracic access. However, transalternative access—largely reflecting TAx procedures—was associated with higher rates of stroke or transient ischemic attack compared with transfemoral access, although overall mortality remained similar between the two strategies [18]. Notably, when considering stroke alone (excluding transient ischemic attack), the rates were comparable between groups [18].

Overall, these observational data support TAx as a viable and effective non‐femoral transarterial strategy for large‐bore structural interventions. However, most of the evidence derives from non‐randomized studies and is therefore subject to selection bias. Nevertheless, the potential for neurologic complications underscores the importance of careful patient selection and meticulous procedural technique.

### 1.4 Percutaneous Versus Surgical TAx

The transition from surgical cutdown to a fully percutaneous strategy has been evaluated in large registry analyses. In a propensity‐matched analysis from the STS/ACC TVT Registry including 4219 patients undergoing Tax TAVR, 1140 underwent percutaneous access and 3079 surgical cutdown. After matching, 30‐day mortality and stroke rates were similar between groups. Percutaneous access, however, was associated with a higher rate of major vascular complications, while life‐threatening bleeding remained similar. Importantly, the percutaneous approach was associated with greater use of conscious sedation and less need for intensive care [27].

A systematic review including 22 studies of large‐bore axillary access for TAVR and Impella found similar 30‐day mortality, stroke, and major vascular complication rates between percutaneous and surgical transaxillary approaches, while major bleeding was significantly lower with the percutaneous strategy [28].

Taken together, the available evidence suggests that pTAx access can reproduce the efficacy of surgical exposure while enabling a more minimalist strategy, at the cost of a learning curve and a continued need for rigorous access‐site management.

### 1.5 Comparison With Other Alternative Access Routes

A large network meta‐analysis including more than 30,000 patients demonstrated that non‐femoral transarterial alternative access routes—such as transaxillary, transcarotid, and transcaval—are associated with clinical outcomes comparable to transfemoral TAVR, whereas transthoracic approaches (transapical and transaortic) consistently show increased short‐ and long‐term mortality [29]. These findings support a contemporary paradigm favoring non‐femoral transarterial strategies whenever transfemoral access is not feasible.

Within the spectrum of non‐femoral transarterial options, each access route presents specific advantages and limitations. TAx, particularly when performed percutaneously, is one of the most widely adopted alternative strategies, owing to its reproducibility, widespread operator experience, and relatively low material costs [18]. Moreover, recent technical refinements—including the use of local anesthesia and radial‐based secondary access—enable real‐time neurologic monitoring and facilitate bailout interventions, further improving procedural control and safety [8, 10]. Registry data also suggest that TAx is associated with shorter hospital stay and lower mortality compared with historical transthoracic approaches, reinforcing its role as a less invasive alternative [19].

However, the main limitation of pTAx remains the relatively higher incidence of neurological complications. In a propensity‐weighted multicenter analysis comparing transcaval and transaxillary TAVR, stroke and transient ischemic attack were significantly more frequent following TAx, whereas bleeding, vascular complications, length of stay, and survival were similar between groups [30]. Similarly, a large network meta‐analysis including more than 43,000 patients reported a higher risk of stroke with trans‐subclavian/TAx compared with transfemoral and transcaval approaches, as well as a higher risk of major or life‐threatening bleeding compared with transfemoral access [31]. These observations need to be interpreted cautiously, given the non‐randomized nature of these studies that may introduce relevant selection bias. Ideally, a randomized comparison between various non‐femoral transarterial strategies could help resolve remaining concerns about the relative safety of different access strategies.

Collectively, these findings suggest that while TAx represents a versatile and widely adopted non‐femoral transarterial strategy, its neurological risk profile warrants particular attention. Accordingly, a structured step‐by‐step approach is proposed to mitigate complications and improve safety in this inherently high‐risk patient subset [8, 10].

## 2 TAx for MCS

TAx has been increasingly adopted for temporary MCS, particularly for Impella (Abiomed, Inc., part of Johnson & Johnson MedTech, USA) implantation. Compared with femoral large‐bore access, the axillary route may facilitate ambulation and rehabilitation, while potentially reducing complications related to prolonged immobilization, including deconditioning and access‐site infection [28, 32, 33]. Accordingly, TAx represents an attractive option in selected patients requiring prolonged MCS support [4, 28, 34] or in patients with unsuitable femoral anatomy requiring a high‐risk percutaneous coronary intervention (HR‐PCI) with the aim of achieving a safe and more complete revascularization [35, 36].

Prospective data from the Axillary Access Registry to Monitor Safety (ARMS), a multicenter observational study including 102 patients, further supports the feasibility of this approach. Successful device implantation was achieved in 98% of cases, with acceptable rates of bleeding complications (approximately 10%) and a low incidence of stroke (1%) [34]. Access‐site management was predominantly percutaneous, with hemostasis achieved using closure devices in most cases, with stent grafting or balloon tamponade as preferred bail‐out solutions. Notably, longer duration of support was associated with an incremental risk of vascular complications, highlighting the importance of careful access‐site monitoring in prolonged MCS support [34].

Registry data from Japan, including 2564 patients treated with Impella for cardiogenic shock, showed that approximately 6.5% received support through a transaxillary or trans‐subclavian route. Despite a distinct higher‐risk clinical profile, 30‐day mortality was comparable to the transfemoral group, supporting the feasibility of this alternative in selected patients [37].

For MCS, the access strategy differs somewhat from TAVR. Sheath dwell time is often longer, delayed closure is common, and hemostasis may need to be obtained after prolonged arterial instrumentation. Accordingly, preserving bailout options and anticipating access‐site bleeding are particularly important in this population [38].

## 3 Procedural Technique

The procedural workflow and the main technical steps of pTAx are summarized in the Graphical Abstract.

### 3.1 Pre‐Procedural Planning

Careful patient selection and pre‐procedural imaging are key for successful pTAx. Multidetector computed tomography (CT) is typically used to evaluate vascular anatomy, including vessel diameter, calcification, tortuosity, and the relationship between the axillary artery and surrounding structures. Pre‐procedural imaging also allows identification of the optimal puncture site and facilitates planning of the fluoroscopic projection required for safe arterial access.

The axillary artery is divided into three segments based on its anatomical relationship to the pectoralis minor muscle; additional important landmarks include the lateral thoracic and the thoraco‐acromial arteries (Figure 1). The preferred puncture site for TAx access is at the transition between the proximal and middle segments of the axillary artery. Distal puncture should be avoided due to the close relationship between the vessel and the brachial plexus, which increases the risk of neurological injury. Conversely, an excessively proximal puncture may increase the risk of pleural injury, pneumothorax, and hemothorax given the proximity of the thoracic cavity. Importantly, access to the middle segment requires traversal of the pectoralis minor muscle; therefore, careful preparation of the muscle plane is essential to facilitate safe arterial puncture and the advancement of large‐bore devices. Avoiding excessively medial, noncompressible punctures and too distal punctures that may increase the risk of brachial plexus injury [4, 7, 32].

**Figure 1:**
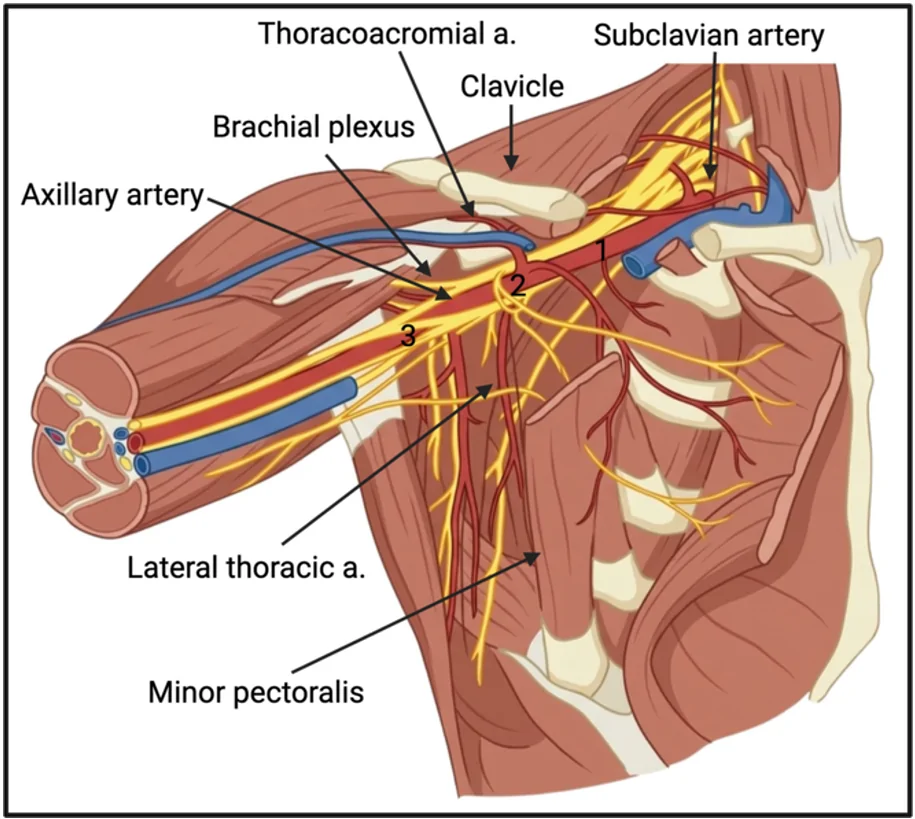
Axillary artery anatomy relevant for percutaneous transaxillary access. The artery is divided into three segments based on its relationship to the pectoralis minor muscle: the proximal segment medial to the muscle, the middle segment posterior to it, and the distal segment lateral to it. Understanding these anatomical landmarks is essential for identifying the optimal puncture zone and avoiding neurovascular complications. [Color figure can be viewed at wileyonlinelibrary.com]

The left axillary artery is generally preferred for TAx. This approach avoids crossing the origin of the right common carotid artery, which may theoretically reduce the risk of cerebral embolization and stroke. In addition, left‐sided access often provides a more favorable trajectory for device delivery. When the aorta is not vertically oriented, the delivery system introduced from the left axillary artery tends to align along the outer curvature of the aortic arch, facilitating a more coaxial interaction with the aortic annulus. In contrast, right‐sided access may direct the delivery system toward the inner curvature of the arch, potentially resulting in a less optimal alignment with the annular plane. A minimal vessel diameter of approximately 6 mm is generally recommended for large‐bore sheath insertion, although contemporary series have reported successful TAx‐TAVR with lumen diameters just above 5 mm in selected patients and with sheathless or lower‐profile systems [39, 40].

Particular attention should be paid to patients with previous coronary artery bypass grafting using a patent left internal mammary artery (LIMA) [41]. A patent LIMA graft is generally considered a relative contraindication to left TAx because large‐bore sheath insertion may compromise graft flow or cause injury at the mammary origin. When feasible, the contralateral axillary artery should therefore be preferred. If left‐sided access is required, careful CT assessment of the vessel diameter at the mammary take‐off is essential, with suggested thresholds ranging from approximately 5.5 to 7.5 mm depending on sheath size and device profile. As an additional maneuver to preserve graft flow, the delivery sheath may be withdrawn distal to the LIMA origin once the device has been advanced into the ascending aorta.

With specific regard to device selection in pTAx TAVR procedures, meticulous preprocedural planning is required, taking into account not only the nominal sheath size relative to vessel diameter but also the effective outer diameter of the delivery system during valve advancement. In balloon‐expandable platforms, 14–16 Fr recollapsible introducer sheaths may transiently expand to approximately 23–24 Fr during transcatheter heart valve passage, a factor that should be carefully considered given the relative vulnerability of the subclavian–axillary arterial segment to vascular injury. Indeed, as the axillary and subclavian arteries are elastic arteries, the lack of a thicker and more fibrous adventitia, such as the femoral artery, predisposes to an increased risk of vascular complications such as rupture or dissection [7]. Furthermore, although TAVR implantation with self‐expanding valves can be performed using a sheathless technique, the use of a larger introducer sheath may be preferred when pre‐dilatation, post‐dilatation, or both are required. For example, the delivery system of the Evolut FX 23–29 mm (Medtronic, Ireland) typically requires an 18 Fr introducer sheath, compared with a 14 Fr access profile when used sheathless. The use of an adequately sized sheath when anatomically feasible may help reduce vascular trauma by avoiding repeated device manipulations across the access vessel when multiple device passages are expected.

Representative cases of pTAx in patients with hostile femoral access are shown in Figures 2 and 3.

**Figure 2:**
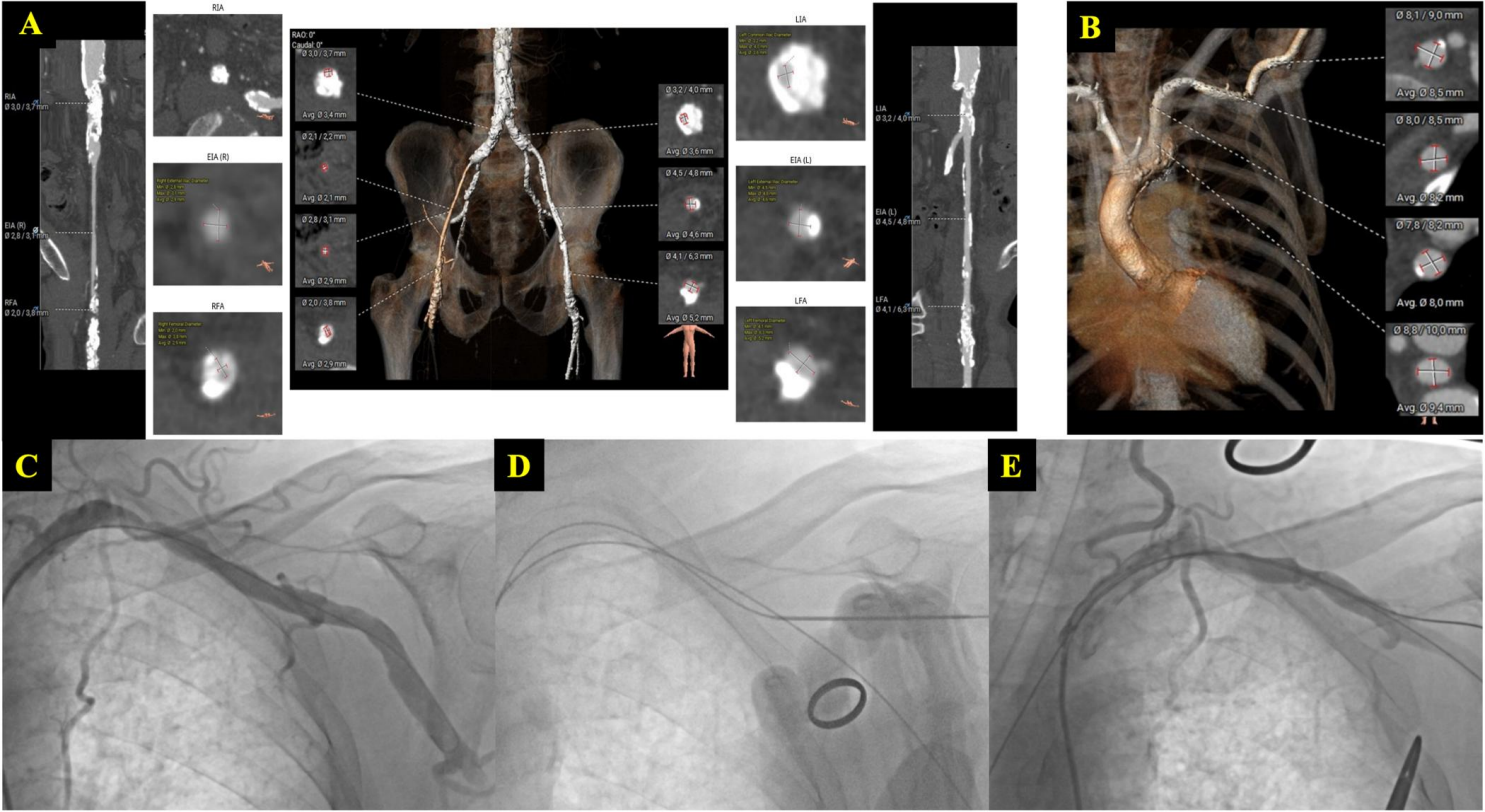
Hostile femoral accesses during transcatheter aortic valve replacement (TAVR). (A) Computed tomography (CT) analysis of left and right aorto‐femoral routes; (B) CT analysis of left subclavian and axillary arteries; (C) Baseline angiography of left subclavian and axillary arteries; (D) Site of puncture wire and a protection wire in place; (E) Hemostasis after closure with two pre‐implanted suture‐based devices. [Color figure can be viewed at wileyonlinelibrary.com]

**Figure 3:**
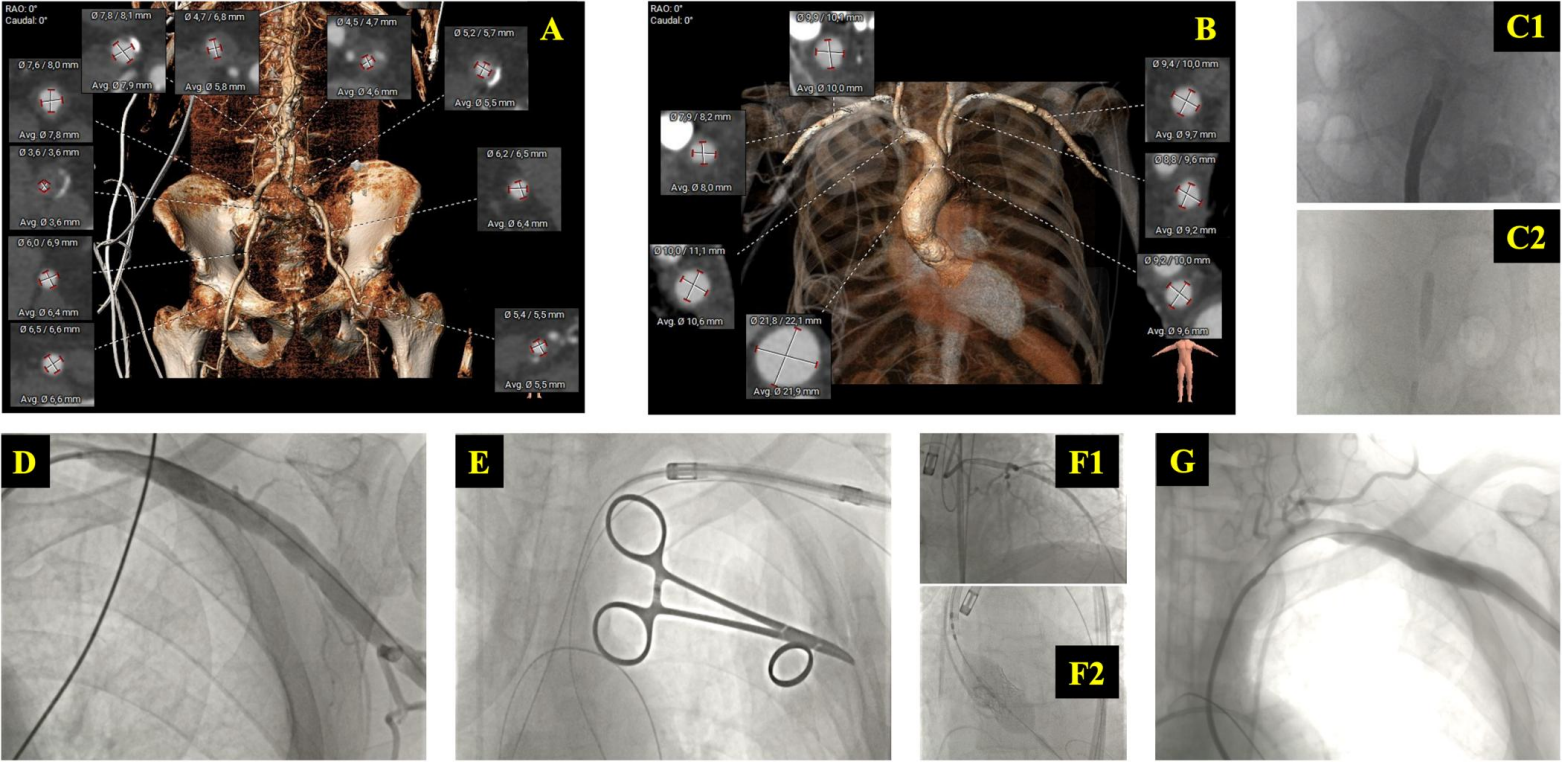
Hostile femoral accesses during transcatheter aortic valve replacement (TAVR) and high‐risk percutaneous coronary intervention (PCI) with mechanical circulatory support in a patient with severely depressed left ventricular ejection fraction (15%). (A) Computed tomography (CT) analysis of left and right aorto‐femoral routes with critical stenosis of right common iliac artery and occlusion of left common femoral artery; (B) CT analysis of both subclavian and axillary arteries; (C1 and C2) Baseline angiography of right iliac artery and balloon angioplasty; (D) Baseline angiography of left subclavian and axillary arteries with protection wire in place; (E) Transaxillary mechanical circulatory support; (F1 and F2) Protected PCI and TAVR; (G) Hemostasis after closure with two pre‐implanted suture‐based devices. [Color figure can be viewed at wileyonlinelibrary.com]

*Practical tips*

- Review CT specifically for vessel diameter, circumferential calcium, tortuosity, and proximity to the vertebral and internal mammary origins.
- Select a puncture site that offers both a straight path for the device, manual compression, and a realistic bailout strategy.
- Avoid heavily calcified anterior wall segments, as they reduce puncture precision and compromise VCD performance.
- Careful selection of the sheath and delivery system per anticipated procedural steps.

## 4 Vascular Access and Puncture Technique

PTAx should be obtained under ultrasound and/or angiographic guidance. A secondary arterial access, obtained via the femoral or (ipsilateral) radial artery, is commonly used for angiographic visualization of the axillary artery and optionally to place a safety wire.

The procedure usually begins with selective angiography of the axillary axis. The ideal puncture site is between the first segment and the second part of the axillary artery, typically just distal to the lateral border of the first rib and proximal to the pectoralis minor muscle. Puncturing the artery over the rib cage provides a stable surface that facilitates compression in case of bleeding, while avoiding too proximal punctures in the subclavian artery or too distal punctures near the brachial plexus.

Under real‐time ultrasound guidance, the artery is punctured with a micropuncture needle at an angle of approximately 30°, followed by insertion of a guidewire and progressive dilation of the access tract. Arm abduction is optional to reduce vessel tortuosity and facilitate advancement of the large‐bore sheath. Once adequate dilation has been achieved, the sheath is advanced into the artery to allow device delivery [13].

It is important to have fast bail‐out options in place. In the original Hamburg experience [7], a safety wire was introduced and snared to create a fast rail, the introduction of balloons and stents. A simpler strategy is to obtain access to the ipsilateral radial artery with a 6 or 7 F sheath [8]. A wire can be easily introduced in case of closure device failure or access site complication.

From a practical standpoint, the procedural setup may also require modification of the catheterization laboratory ergonomics. Monitors may need to be repositioned to the right side of the patient for a left pTAx approach, and it is worthwhile to install an additional monitor at the head side of the patient.

*Practical tips*

- Use ultrasound to confirm a healthy anterior wall, avoid blind puncture in mobile vessel segments, and assist to identify the brachial plexus.
- Maintain ipsilateral radial access to allow rapid re‐access until patent hemostasis is confirmed.

## 5 Device Delivery and Sheath Management

Once large‐bore access is established, the subsequent procedural steps are largely dictated by the intended intervention. For TAVR, pTAx offers a relatively direct route to the ascending aorta and is particularly attractive for self‐expanding valve systems, which use low‐profile, often sheathless or integrated‐sheath delivery systems that track more easily through the axillary artery and benefit from the stable platform required for gradual valve deployment. In recent series, self‐expanding 14–16 F platforms were commonly used through a fully pTAx [26].

For MCS, access‐site management is heavily influenced by device dwell time. The longer the sheath remains in place, the greater the risk of bleeding, hematoma, thrombosis, and local tissue complications. This makes puncture precision, sheath stabilization, and maintenance of rescue access particularly important [13]. Engaging the nursing team through regular procedural training on device management and ventricular support principles is essential to ensure optimal safety in access site care. During prolonged axillary MCS support, meticulous daily inspection of the arteriotomy site is recommended to assess for signs of infection, hematoma, or external bleeding [33]. Additionally, both the catheter and driveline should be secured to prevent traction at the arteriotomy site and to minimize excessive upper extremity movement. Finally, regular assessment of the radial or brachial pulse is essential to detect and prevent ischemic complications of the upper extremity.

*Practical tips*

- Maintain a coaxial trajectory during sheath advancement to reduce wall trauma.
- Be alert to vessel mobility and wire bias when advancing stiff systems.
- Minimize unnecessary exchanges across the supra‐aortic vessels to reduce embolic and neurologic risk.
- Meticulous daily inspection of the arteriotomy site is recommended to assess for signs of infection, hematoma, ischemia or external bleeding in case of prolonged support.

## 6 Closure Strategies and Hemostasis

Whenever feasible, vascular closure is preferably performed in the catheterization laboratory rather than at the bedside [42, 43]. The catheterization laboratory allows fluoroscopic guidance, balloon‐assisted closure, and immediate angiographic assessment of hemostasis and vessel patency, while preserving the possibility of prompt endovascular bailout should bleeding or vessel injury occur.

### 6.1 Pre‐Closure With Suture‐Mediated Devices

Reliable hemostasis is one of the most critical aspects of pTAx. Arteriotomy closure and patent hemostasis should always be confirmed to avoid overt or occult bleeding or occlusion. This can be achieved by fluoroscopy (leveraging the second radial or femoral or ipsilateral radial/ulnar access or by ultrasound. In contemporary practice, the most common strategy is a suture‐mediated pre‐closure using two ProGlide or ProStyle devices (Abbott, USA). A combination of suture‐mediated and collagen‐plug closure (i.e., Angioseal [Terumo, Japan] or MANTA [Teleflex, USA]) has also been described but does not allow preservation of the access if the device fails [13]. The axillary artery differs from the femoral artery in wall composition and may be more vulnerable to “pull‐through” if excessive tension is applied to the sutures. Conversely, successful closure may also compress the vessel, making angiographic verification of patency relevant. Schäfer et al. reported a marked difference in closure performance between ProStar and ProGlide, with substantially better results using ProGlide [7].

In patients requiring prolonged MCS support, pre‐closure sutures can be left untied and secured externally under a sterile dressing, taking care to avoid contamination, entanglement, or inadvertent tightening until definitive closure at device removal.

*Practical tips*

- Favor double suture‐mediated closure for large‐bore axillary arteriotomies.
- Tighten sutures gradually; excessive tension risks arterial wall injury or pull‐through.
- Do not remove the safety wire before confirming hemostasis and vessel patency.
- Prioritize balloon‐assisted dry closure.
- Always confirm patent hemostasis by angiography or ultrasound.

### 6.2 Balloon‐Assisted Dry Closure

Balloon‐assisted or “dry” closure has become one of the most useful adjunctive techniques in pTAx procedures. The principle is to deploy a balloon proximal to the arteriotomy to ensure a blood‐free environment while the large sheath is removed, and the pre‐placed sutures are tightened. This improves visualization and hemostatic control, minimizes blood loss, and preserves time for escalation if closure is incomplete [13]. This balloon can be introduced via a femoral arterial or contralateral radial access.

More recently, an ipsilateral suture‐mediated dry‐closure technique using ipsilateral radial or ulnar access has been described. In this approach, a 6–7 F sheath is inserted in the ipsilateral radial or ulnar artery; after the intervention, a balloon sized 1:1 to the artery is advanced proximally to the arteriotomy and inflated while the two pre‐installed sutures are tied. If hemostasis is incomplete, the balloon can be re‐inflated, and additional closure devices or covered stenting can be performed over the retained rescue wire. In a 77‐patient series, this strategy significantly reduced vascular complications, VCD failure, bleeding, and bailout surgery/stenting compared with the earlier closure approach [8].

A representative case of balloon‐assisted dry closure after pTAx microaxial flow pump support is shown in Figure 4.

**Figure 4:**
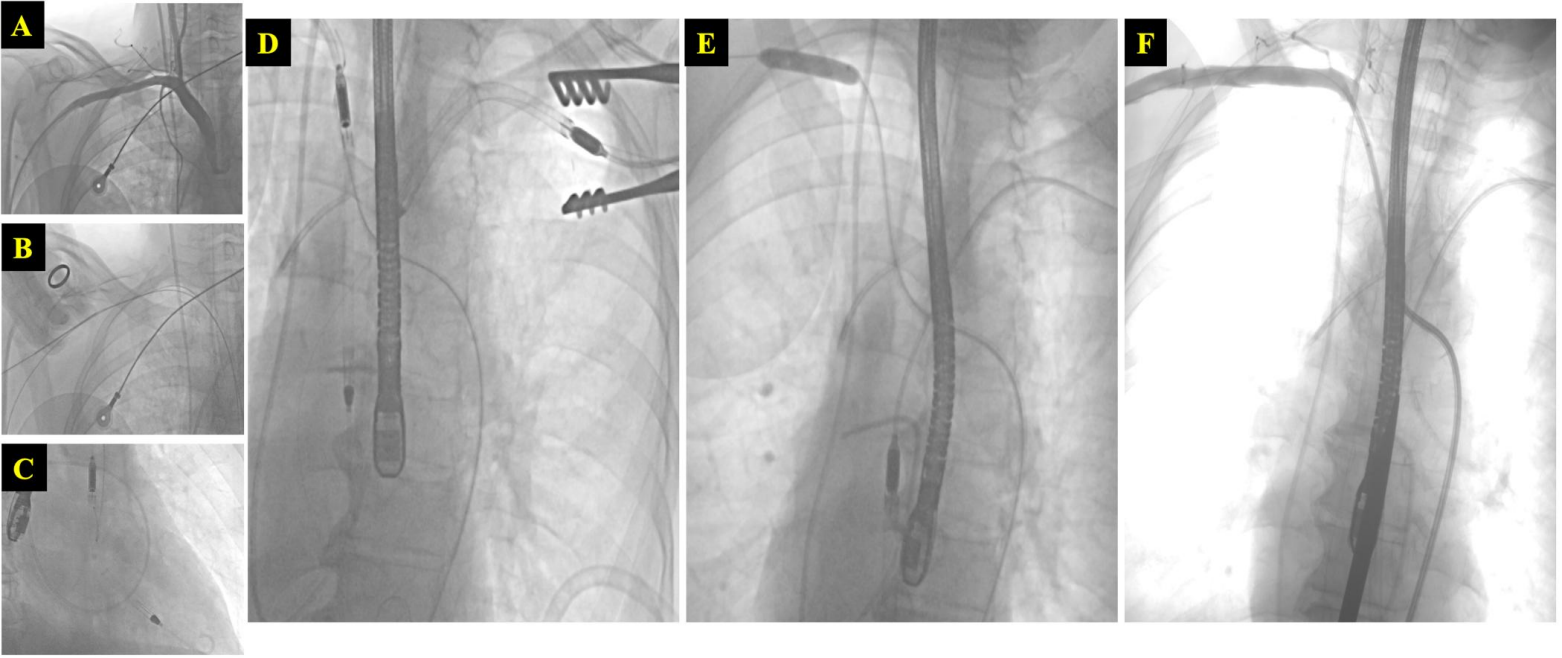
Percutaneous left ventricular support with an Impella CP via right axillary access, with balloon‐assisted dry closure for device removal and simultaneous upgrade to an Impella 5.5 via right surgical subclavian access due to refractory cardiogenic shock. (A) Angiography of the right axillary artery; (B) Percutaneous puncture of the right axillary artery; (C) Impella CP implantation; (D) Removal of the Impella CP and surgical implantation of an Impella 5.5 via left subclavian access a few days later due to refractory cardiogenic shock; (E) Balloon‐assisted dry closure of the right axillary access; (F) Final angiography after dry closure with two pre‐implanted suture‐based devices confirming adequate hemostasis. [Color figure can be viewed at wileyonlinelibrary.com]

*Practical tips*

- Balloon‐assisted dry closure should be standard whenever feasible in large‐bore TAx closure.
- Size the balloon 1:1 to the artery and position it proximal to the arteriotomy before sheath removal.
- Re‐inflate the balloon and be prepared for bail‐out solutions in case of incomplete hemostasis.

### 6.3 Plug‐Based Closure and Hybrid Strategies

Plug‐based closure devices have been used for pTAx, but they do not preserve access if failure occurs. This represents a relevant limitation in the axillary territory, where bailout bleeding control can be more demanding [13].

The Rotterdam experience is particularly informative: their early cohort relied mainly on a plug‐based closure strategy and had higher rates of VCD failure and bailout‐covered stenting. Transition to a double‐suture dry‐closure strategy was associated with better safety outcomes [8].

*Practical tips*

- Plug‐based closure is less forgiving when bleeding occurs.
- If using a plug‐based device, ensure a clear bailout plan with preserved wire access and proximal balloon control.
- Hybrid closure with suture plus adjunctive device may be useful when hemostasis is incomplete.

## 7 Complications and Management

Complications of TAx are distinct from femoral complications because of the vessel's mobility, proximity to the brachial plexus, and relation to major supra‐aortic branches. Bleeding is the most frequent complication, while hematoma, neurologic events, pseudoaneurysm, hand ischemia, nerve injury, dissection, and thrombosis are sporadic [8, 34].

### 7.1 Bleeding and Closure Failure

Bleeding is the dominant hazard of pTAx. Hemostasis failure can result in bleeding into the axillary space, with secondary compression of the brachial plexus and potential permanent neurologic injury. If the initial closure strategy fails, prolonged balloon tamponade should be the first bailout maneuver. If extravasation persists, covered stent implantation is an effective endovascular solution. Schäfer et al. reported that closure failures in the early percutaneous series were managed successfully with endovascular stent graft implantation, thereby avoiding open surgical repair [7]. Self‐expanding covered stents are recommended for better apposition and crush resistance, potentially considering branch coverage, especially near the vertebral and internal mammary arteries [13]. Operators should be familiar with diameter, length, and delivery profile (French size) of available balloon catheters and balloon‐expandable and self‐expanding covered stents on the shelf.

Bailout‐covered stent implantation for severe bleeding after failed suture‐mediated closure is illustrated in Figure 5.

**Figure 5:**
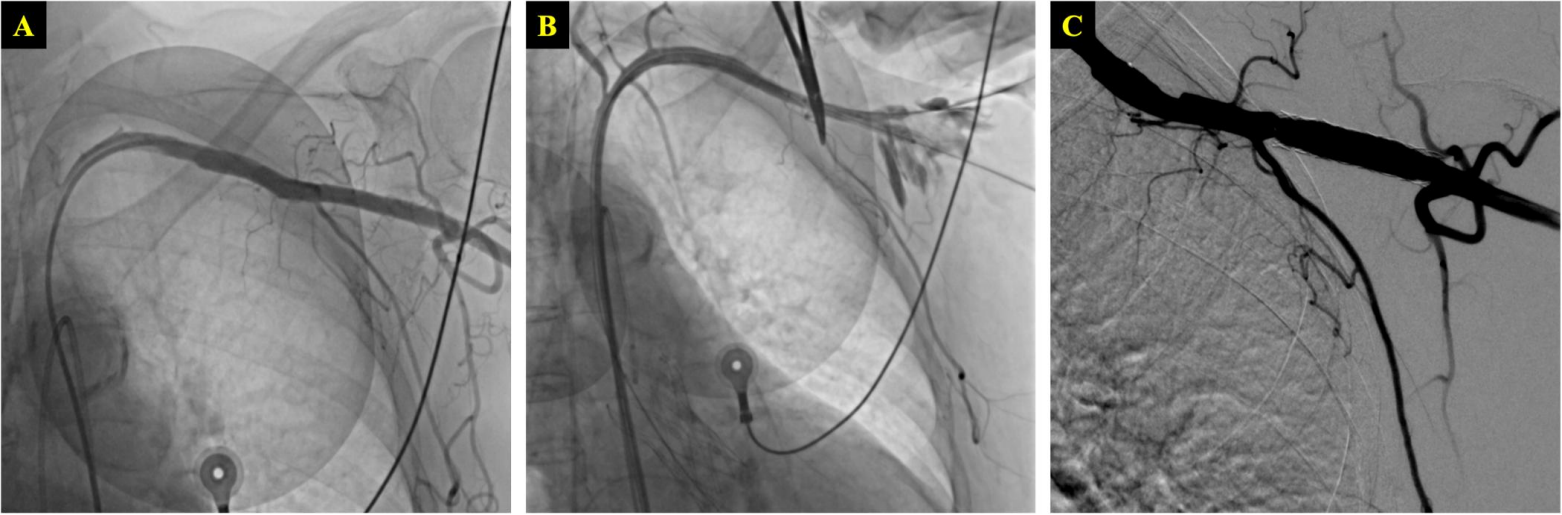
Bailout implantation of a covered stent due to severe bleeding following ineffective percutaneous suture‐based closure. (A) Angiography of the subclavian and axillary arteries prior to percutaneous puncture; (B) Angiography demonstrating severe bleeding after closure with two pre‐implanted suture‐based devices; (C) Final angiography following covered stent implantation showing adequate hemostasis. [Color figure can be viewed at wileyonlinelibrary.com]

*Practical tips*

- Stepwise approach: balloon tamponade first, covered stent second, surgery third.
- Keep access options for stent delivery in mind: femoral, ipsilateral brachial, or radial/ulnar depending on anatomy and device profile.
- A short, covered stent may fail to seal; an overly long one may jail important branches.

### 7.2 Hematoma, Pseudoaneurysm, and Nerve Compression

Because the axillary space is not directly visible, a significant hematoma may develop before it becomes clinically evident. Moreover, pseudoaneurysms may require endovascular or surgical treatment depending on size and symptoms [13, 44].

The brachial plexus is particularly vulnerable in this setting. Both expanding hematoma and aggressive manual compression may contribute to neurologic injury. This is one reason why dry closure and controlled balloon‐assisted hemostasis may be preferable to forceful manual compression in large‐bore cases.

*Practical tips*

- Check for patent hemostasis after use of closure devices.
- Maintain clinical vigilance for occult bleeding.
- New arm pain, paresthesia, or weakness should prompt suspicion for a compressive hematoma.
- Avoid prolonged aggressive compression unless proximal balloon control is in place.

### 7.3 Stroke and Neurologic Events

Stroke is a major complication after TAx TAVR in comparative studies [21, 25, 45]. Mechanisms are likely multifactorial and may include catheter manipulation in the aortic arch and subclavian artery, embolization from local atheroma, or interaction near supra‐aortic branch ostia. The higher stroke rates reported in the UK registry and comparative analyses versus transcaval or TF access reinforce the need for careful device manipulation and procedural simplification whenever possible [25]. In order to minimize the risk of cerebrovascular events, preprocedural CT imaging should be performed to characterize relevant anatomical features, including severe tortuosity, mobile or ulcerated plaques, and calcification of the aortic arch or subclavian axis. In addition, a thorough neurological assessment and detailed clinical history are essential to identify patient‐specific risk factors for neurological events, such as advanced age, prior stroke, severe PAD, carotid artery disease or prior carotid intervention, and a high burden of diffuse aortic arch atherosclerosis [45, 46, 47].

*Practical tips*

- Minimize catheter exchanges and avoid unnecessary manipulation near the vertebral artery origin.
- Use the most direct and stable wire path possible.
- Reduce procedural time and arch instrumentation when feasible.
- Use full anticoagulation during the procedure and avoid protamine whenever possible.
- Review CT for aortic arch tortuosity, mobile or ulcerated plaques, or severe calcification.
- Review history of previous neurological events, severe carotid disease, and diffuse PAD.

### 7.4 Thrombosis and Ischemic Complications

Prolonged dwell time of large‐bore devices in the axillary artery is associated with an increased risk of device‐ or sheath‐related thrombosis, even under systemic anticoagulation. This phenomenon is largely time‐dependent and may remain clinically silent due to the extensive collateral circulation of the shoulder, which often preserves distal limb perfusion. Nevertheless, thrombus formation may lead to distal embolization or access‐site complications requiring endovascular treatment [13, 48].

*Practical tips*

- Reassess the need for support daily to minimize dwell time.
- Ensure adequate anticoagulation during device support.
- Consider angiographic control at explant.
- Be prepared for endovascular bailout (thrombectomy/angioplasty).

### 7.5 Infections

Access‐site infection after pTAx is uncommon and is reported less frequently than after transfemoral large‐bore strategies. This is likely related to the more favorable soft‐tissue environment of the axillary region and the absence of groin contamination [13, 49]. In the setting of prolonged MCS, these characteristics represent an important advantage of axillary access compared with femoral cannulation, which may be associated with higher risks of local infection, immobility, and patient deconditioning.

*Practical tips*

- Maintain sterile dressing and secure device fixation during prolonged support.
- Inspect the axillary insertion site daily for signs of infection.
- Early mobilization may reduce local complications during prolonged MCS.

## 8 Conclusion

pTAx has become a valuable alternative to transfemoral large‐bore access with acceptable safety in patients with hostile iliofemoral anatomy undergoing TAVR or temporary MCS. Neurological events and access‐site bleeding remain important challenges. Safe implementation depends on meticulous imaging‐based planning, precise ultrasound‐ and angiography‐guided puncture, incorporation of bailout options, careful suture‐based closure and confirmation of patent hemostasis after arteriotomy closure.

## Funding

The authors have nothing to report.

## Conflicts of Interest

G.G.S. has received speaker fees from Medtronic, Abbott, Biosensor, Meril, Johnson & Johson; he is proctor for Meril Medical and Biosensor. M.L. reports speaker fees from Philips and consultancy fees from Boston Scientific. R.V. is consultant for Abbott Vascular and Medtronic; received lecturing fees from Abbott Vascular, Abiomed, Daiichi Sankyo, Edwards Lifesciences, Medtronic, Novartis, Novo Nordisk, Philips, and SIS Medical; and served as member of advisory boards for Amarin and Amgen. G.P. reports speaker and/or consultancy fees from Amgen, Sanofi, Novartis, Daiichi Sankyo, Amarin, Aurora BioPharma, Malesci, PIAM, Boehringer Ingelheim, Bayer, Pfizer/BMS, AstraZeneca, Biotronik, Terumo, Medtronic, Abbott, Edwards, Amicus, Novo Nordisk, and Chiesi. The other authors declare no conflicts of interest.

## Data Availability

The authors have nothing to report.
